# Chondrosarcoma of the Ribs

**DOI:** 10.7759/cureus.9158

**Published:** 2020-07-12

**Authors:** Muhammad Tahir, Jawaria Rahman, Hassan Arekemase, Tayyaba Zubair, Abdul Basit

**Affiliations:** 1 Pathology, Case Western Reserve University School of Medicine, Cleveland, USA; 2 Pathology, City of Hope, Comprehensive Cancer Center, Monrovia, USA; 3 Anatomical and Clinical Pathology, Saint Barnabas Medical Center, Livingston, USA; 4 Internal Medicine, Coney Island Hospital, Brooklyn, USA

**Keywords:** chondrosarcoma, periosteal chondrosarcoma, primary neoplastic bone tumor, chondrosarcoma of rib

## Abstract

Chondrosarcoma is a unique kind of tumor that originates from the cartilage-producing neoplastic mesenchymal cells and appears in both the appendicular and atlantoaxial skeleton. It is the second most prevalent neoplastic bone tumor, with an occurrence of 0.79/100,000/year. The biological presentation of this cancer fluctuates extensively, depending on the grade and anatomical location. Since chondrosarcoma is predominantly resistant to conventional chemo- and radiation therapy, surgical resection remains the sole curative treatment, although at present new treatment modalities are under investigation.

## Introduction

Chondrosarcoma (CS) is the second most frequent primary solid neoplastic tumor of the bone after osteogenic sarcoma [1]. It is a unique type of neoplasm that generally originates from the bones, but it can occasionally appear in the soft tissue near the bones [2]. CS may occur de novo or develop from bony exostosis or subsequent radiotherapy [3,4]. It is distinguished by the development of hyaline cartilaginous malignant tissue [1]. The majority of the most common locations where CS tumors can be found are the shoulder, pelvis, hips, and thoracic wall. Furthermore, uncommonly the base of the skull is affected [2]. Regarding its occurrence on the chest wall, it is frequently identified in the anterior chest wall, in the superior five ribs, and in close proximity to the costochondral junction [5].

The patients with CS generally complain of and present signs and symptoms of swelling or palpable mass with increasing pain and fracture due to weakened bone [2]. CS roughly accounts for three new cases per 106 population per year [6]. CT with intravenous contrast is the gold standard radiographic study for diagnosis and operative purposes [7,8]. Since CSs do not react well to chemotherapy or radiotherapy, the treatment of choice is a surgical treatment with early diagnosis and radical excision with widely negative microscopic margins at the first operation [9]. The 10-year survival rate after wide resection is 96.4% as compared with 65.4% for those who underwent local excision only [10].

## Case presentation

Here we report the history, diagnostic evaluation, and management of a patient presenting with anterior chest wall mass pathologically identified as CS of the ribs.

A 45-year-old male, with weight of 74.5 kg and height of 178 cm, presented with an enlarged anterior chest wall solid mass just lateral to the sternum. CT imaging confirmed expansile osseous lesion with cortical destruction arising from the costochondral junction involving the fifth rib with extension up to the fourth and sixth ribs. No metastatic disease was found on chest CT, and PET scan confirmed no distant disease. Biopsy revealed CS, and radical resection procedure was performed.

**Figure 1:**
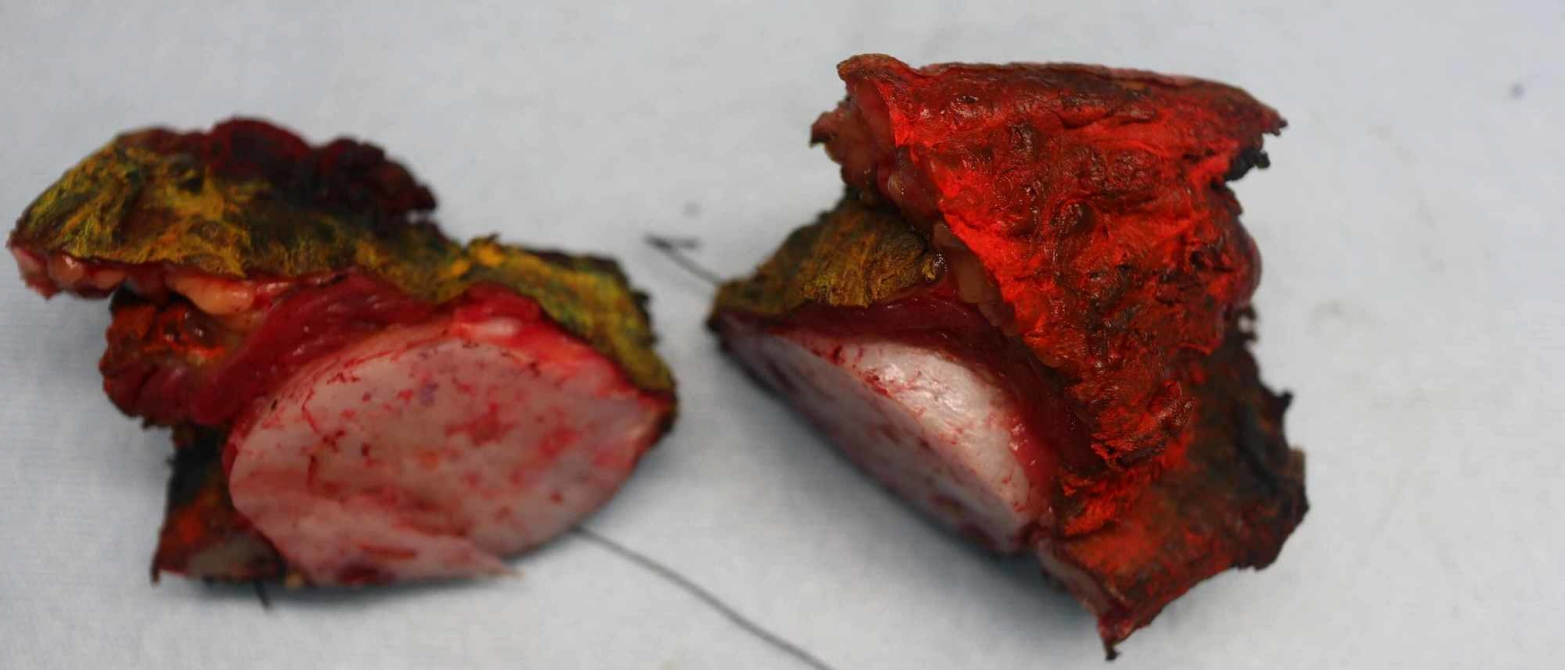
Gross surgical specimen.

**Figure 2:**
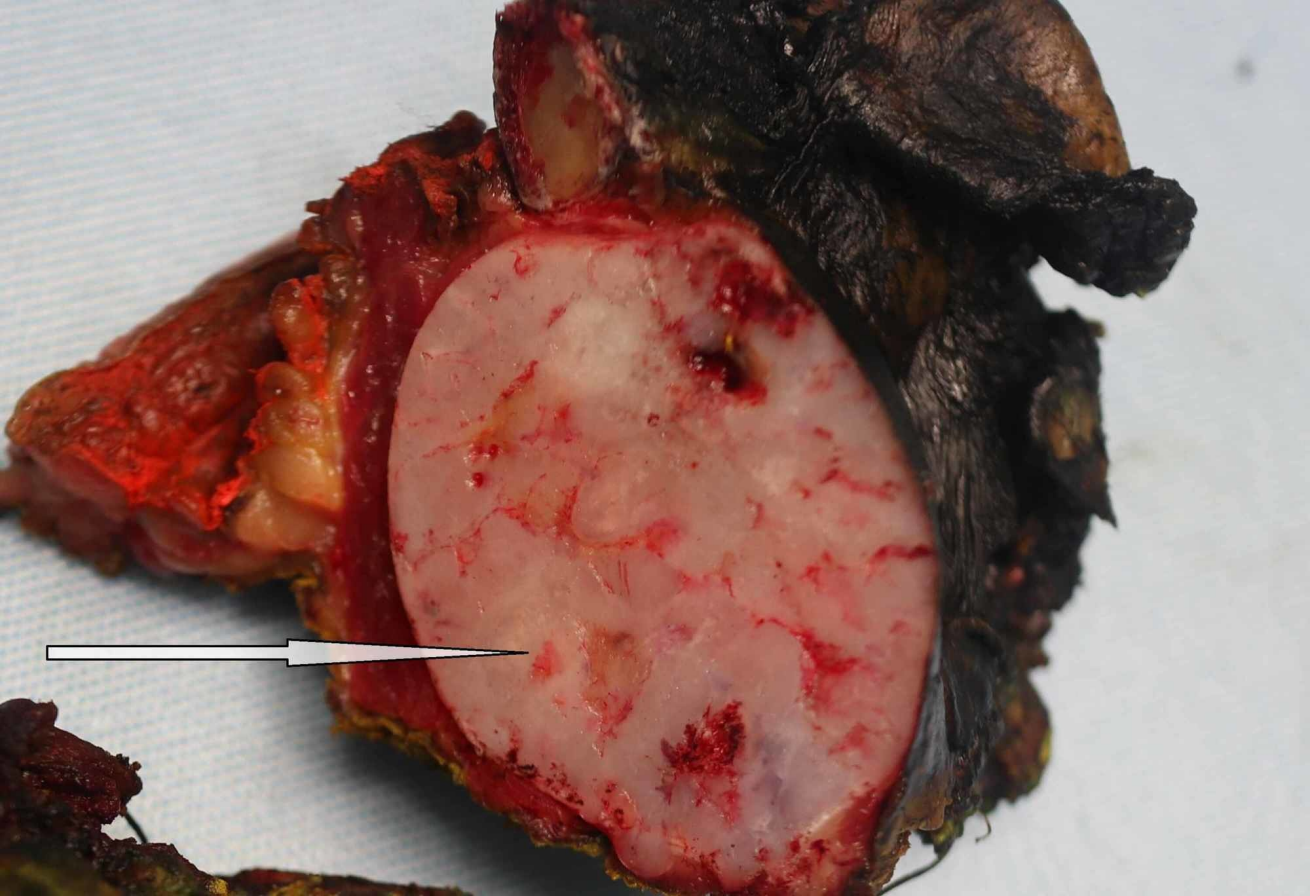
Gross surgical specimen, with the arrow indicating the tumor.

Gross pathological examination of the specimen revealed a fragment of the ellipse of skin, soft tissue, and bone measuring 9.0 x 7.5 x 6.0 cm (Figure 1). Cut section revealed a round well-circumscribed, white/yellow-tan cartilaginous firm mass measuring 5.0 x 4.0 cm. Patchy areas of hemorrhage were identified. No necrosis was present. The lesion was more than 1 cm from all margins (Figure 2).

**Figure 3:**
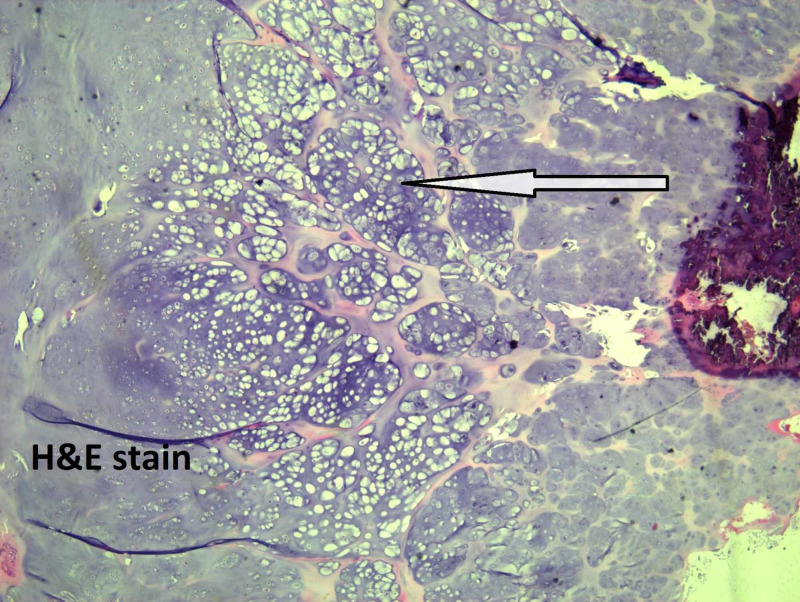
Atypical chondrocytes.

**Figure 4:**
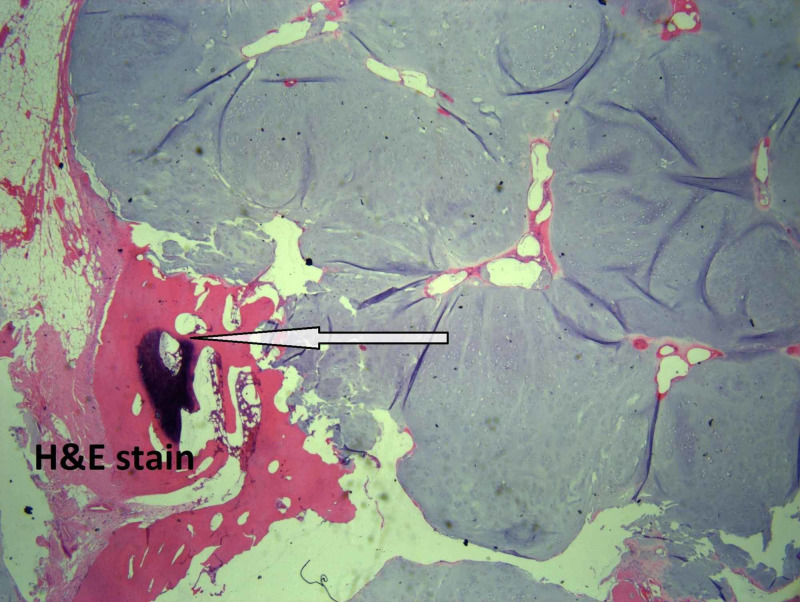
Bone invasion and restoration.

**Figure 5:**
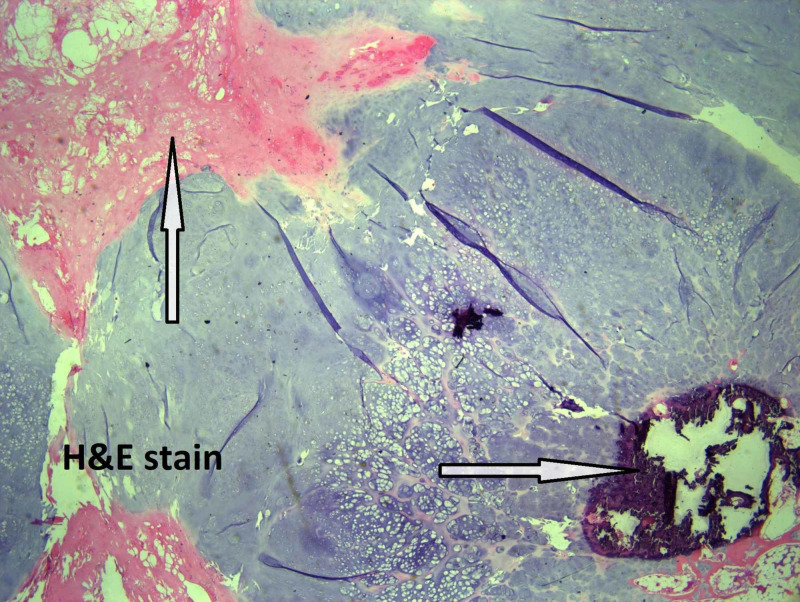
Myxoid degeneration with bone formation.

**Figure 6:**
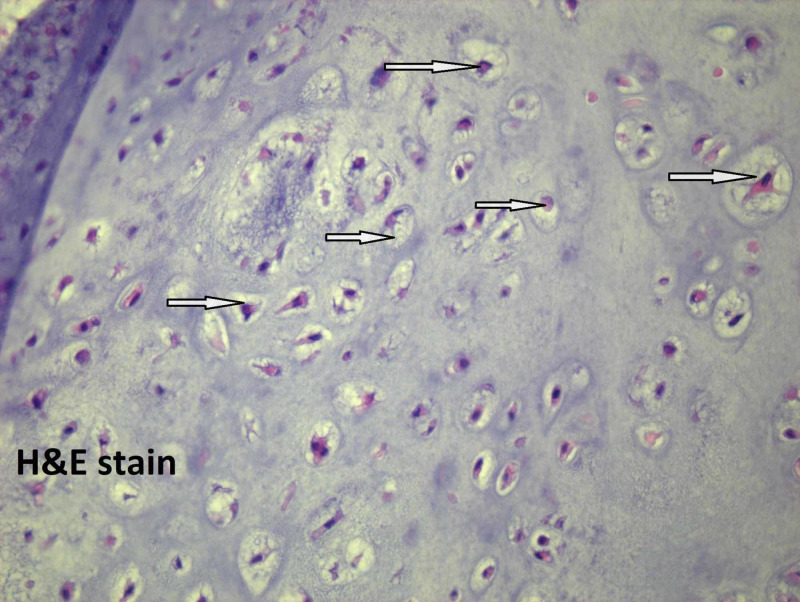
High-power microscopic examination showing atypical chondrocytes.

**Figure 7:**
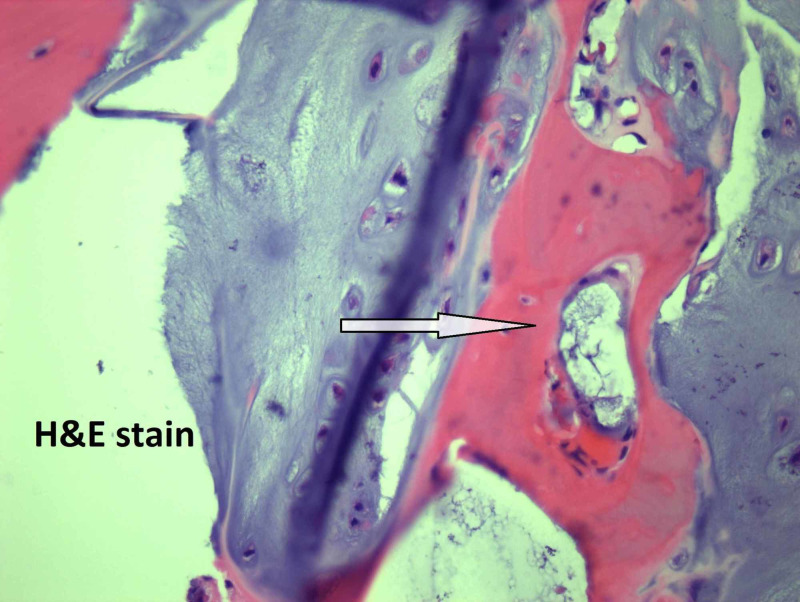
Bone invasion.

Microscopically, the tumor size is 5 cm, the mitotic rate is 1/10 HPF, and well-differentiated low-grade chondrosarcoma with staging pT1, pNx is present. On microscopic examination, atypical chondrocytes (Figure 3), bone invasion and restoration (Figure 4), and myxoid degeneration with bone formation (Figure 5) can be seen. On high-power microscopic examination of H&E-stained slides, atypical chondrocytes (Figure 6) and bone invasion (Figure 7) can be seen.

## Discussion

Chondrosarcoma is a combined title for a cluster of heterogeneous, usually slow-growing, primary neoplastic tumors of the bone [1]. More than 90% of CSs are conventional CSs. Roughly 90% of these are low-to-intermediate grade (grade 1-2) and react indolently and rarely metastasize [11]. Only 5-10% of CSs are grade 3 and have high metastatic potential [12].

CSs that arise unrelated with a prior lesion are called primary CSs, whereas the secondary ones develop from a pre-existing benign cartilage tumor such as an osteochondroma or enchondroma. Additionally, they are classified as central when they originate within the medullary cavity and as peripheral when they grow from the bone surface, e.g., the cartilage cap of an exostosis. Primary CS is almost consistently central, whereas secondary CS can be central or peripheral [13].

The neoplastic primary bone tumor is made up of chondrocytes with varying degrees of malignancy (Table 1). CSs are broadly classified as follows:

Ø Primary CSs, which include the following:

· low- and high-grade dedifferentiated CSs

· clear cell CSs

· mesenchymal CSs

Ø Secondary CSs, which originate from benign cartilage lesions including the following:

· osteochondromas (<1% risk of malignant transformation)

· multiple hereditary exostoses (1-10% risk of malignant transformation) 

· enchondromas (1% risk of malignant transformation)

· Ollier's disease (25-40% risk of malignant transformation)

· Maffucci syndrome (100% risk of malignant transformation)

**Table 1:** Classification of bone tumors.

Benign	Malignant
Chondroma	Osteosarcoma
Osteochondroma	Chondrosarcoma
Chondroblastoma	Fibrosarcoma
Enchondroma	Plasmacytoma
Osteoblastoma	Ewing’s sarcoma
Periosteal chondroma	Lymphoma
Osteoid osteoma	Malignant fibrous histiocytoma

Variants of chondrosarcomas

Periosteal Chondrosarcoma

This is an uncommon type accounting for 1-2% of all cases of CS [14]. The development of the tumor starts at the outer surface of the bone (for the most part the metaphysis of the distal femur or proximal humerus) and arises in the soft tissues as a lobulated mass. The cortex is rarely typical, either eroded or frequently thickened by the tumor, yet never decimated. Ring-like calcifications can be spread or restricted inside the mass. Medullary contribution assessed on CT or MRI is uncommon and limited. The finding of periosteal chondroma can be achieved by histology alone. Most of the patients are young, and lesions are usually small without any pain and are found all the more distally on the skeleton. The result is commonly favorable after a suitable careful resection.

Mesenchymal Chondrosarcoma

This type, accounting for 2-3% of all CSs, consists of an undifferentiated cell component with well-differentiated cartilaginous regions [15]. Usual skeletal locales are the ribs, femur, vertebrae, and pelvic bones. Around one-third of cases include the extraosseous sites: soft tissues, brain, or meninges. The prognosis is bad, with rapid metastasis to lymph nodes, lungs, and bones. The tumors are massive, vigorous, and damaging lesions, basically have a lytic pattern. Multi-drug chemotherapy utilized in osteosarcomas can be combined with radiotherapy and surgery; however, the 10-year survival is just 28% [16].

Clear Cell Chondrosarcoma

An uncommon type (2%) of CS, these lesions are differentiated by their cytology, epiphyseal area in long bones, and sluggish growth [17]. The most affected patients are males in their third to fifth decade of life, with swelling and pain with the most common clinical symptoms. One-fourth of the cases have been accounted for as pathological fractures but few are incidental findings. The most typical sites involved are the humerus, tibia, and femur. A geographic lytic epiphyseal lesion with extension to the metaphysis is the special characteristic of this low-grade tumor. The margins are usually well-defined, but indistinct or sclerotic margins have also been reported. CT might be helpful in delineating lobulated edges and calcified lattice. MRI depicts a well-differentiated low signal on T1-weighted images and heterogeneous high signal on T2-weighted images. Radical surgery is the treatment of choice. The prognosis is favorable with a five-year survival of 92%, even though metastases are found in 15% of cases (bones, lungs, and brain) [18].

Dedifferentiated Chondrosarcoma

This form accounts for 10-12% of all CSs. It is described by unique histology and a very unfavorable prognosis. The biphasic tumor correlates low-grade CS with a non-chondroid high-grade sarcoma. This tumor is very destructive, especially the metaphyseal or diaphyseal variants. The osteolytic lesion may predominate, but most of the lytic area is associated with calcifications, leading in the bi-morphic pattern. A giant soft tissue mass without calcifications seen on CT or MRI additionally demonstrates this diagnosis. Imaging assists in coordinating biopsy of the lytic area to improve the histological finding. The treatment includes adjuvant chemotherapy or radiotherapy and surgery. The prognosis is very bad, with an overall five-year survival rate of only 8.5-13% [18].

Secondary Chondrosarcoma

Of all CSs, 12% are developed in a pre-existing lesion. As its name indicates, it may be secondary to a solitary osteochondroma, Paget’s disease, osteochondromatosis, enchondromatosis (Ollier’s disease), fibrous dysplasia, irradiated bone, or synovial chondromatosis [18]. The presence of an extending exostosis related to pain, less mineralized zone in the cartilage cap, and calcifications in the soft tissues with thickening of the cap (>1 cm) on CT and MRI suggest sarcomatous transformation [19].

Considerable new insights into the molecular cell biology have been growing in the ongoing past. Cytogenetics and immunohistopathology contribute to a deeper understanding of the nature of CSs at the molecular level, which will potentially lead to a better clinical understanding and likely to better-targeted care [20].

## Conclusions

Although surgery is an effective treatment option for CS, it is essential to prepare a secure surgical field and perform reconstruction by considering the location, resection, onset, and re-occurrence rate. Due to the possible likelihood of late local and systemic relapse, patients who were treated by surgical resection should undergo standard surveillance and screening for their whole life. Surveillance should comprise physical examination and thoracic imaging with either posterior-anterior/lateral chest radiograph or CT scan every three to six months for the first 5 years and afterward annually for a minimum of 10 years.
